# PPAP: Perspective Projection Augment Platform with Pan–Tilt Actuation for Improved Spatial Perception

**DOI:** 10.3390/s19122652

**Published:** 2019-06-12

**Authors:** JungHyun Byun, Tack-Don Han

**Affiliations:** Department of Computer Science, Yonsei University, Yonsei-ro 50, Seodaemun-gu, Seoul 03722, Korea; junghyun.byun@msl.yonsei.ac.kr

**Keywords:** projection mapping, spatial augmented reality, virtual depth perception, human–computer interaction

## Abstract

In this paper, we propose PPAP, an augmented reality platform with an actuated projector for dynamic user-perspective projection. In PPAP, a stationary camera is used jointly with a pan–tilt motorized projector-camera unit. With the servo control of the steerable pan–tilt system, the system is able to continuously orient itself to match the user’s view center of the projection-mapped surface. This provides users with greatly widened viewing angles in the augmented scene, when compared to the stationary projection. Through user studies, in which users judged the size and distance of a projected virtual object, we verified that the perspective projection with the actuated projector helps users better understand the spatial relationship of the virtual object in the augmented scene in terms of depth perception.

## 1. Introduction

Augmented reality (AR) is in essence the art of superimposing the computer-generated graphics onto the real world, thus merging both worlds. Thus, sometimes called mixed reality (MR) or extended reality (XR) in a broader sense, AR depicts its unique characteristic compared to virtual reality (VR). In a VR setup, a user is typically completely cut off from the real world by blocking the non-screen peripheral area of a head-mounted device (HMD). In an AR environment, conversely, a user has to remain in the physical world, and virtual contents should augment, not replace, real objects. Consequently, the portion of the real world merged with the virtual world–field of view (FOV), viewing angle or interaction area in other words—heavily affects the quality of experience and sense of immersion.

Bimber and Raskar in [1] grouped AR devices or environments into three categories by their display characteristics and locations as (1) head-mounted (head-attached), (2) mobile (hand-held) and (3) spatial AR (SAR). Recently, several HMD or mobile device (such as a smart phone)-based AR experiences have been introduced, such as Microsoft HoloLens or Apple’s ARKit. Popular as they may be, however, both types of AR devices have innate limitations of being too heavy or cumbersome for users to wear or carry all the time during long-hour usage. In addition, due to limitations in the display technology and the screen size, the effective FOV that users perceive leave room for much improvement, hindering a fully immersive AR experience.

On the other hand, SAR, which is typically implemented with projection mapping on the real world surface, demonstrates the unique characteristic that the device and display are detached from the user. This characteristic of projection-based SAR, or just projection AR, offers distinctive advantages over other types of AR. As the display device is separated from the user, a minimal or even no user instrument at all is required, allowing a more comfortable, and thus immersive and longer AR experience.

The detachment of the device also grants a much wider field of view of the user in the AR environment. A projector like any other display technologies has its own limited FOV. However, depending on the installation or configuration of the projector(s), the effective FOV that a user perceives may greatly differ. For example, a projector can be simply installed far away from the wall to increase its projection surface area. In addition, a number of projectors can be installed in combination to create unified immersive projection mapping. CAVE (Cave automatic virtual environment) itself [2] and CAVE-like environments, such as RoomAlive [3], are prominent examples of this immersive projection AR setups. In an CAVE-like environment, generally multiple projectors and cameras are installed around a cubic space, where four (front, right, left, floor) or more faces out of six are fully covered with projection graphics. With every surface mapped with projection images, the room itself is converted into an AR space where the effective FOV can overcome the FOV limitation of a single physical projection device.

However, if we closely examine the examples of the CAVE-like environments, other drawbacks of the AR environment setup are noticeable that could negate the initial benefits of projection AR. The foremost limitation is the excessive number of the devices needed to configure an AR space. To install a minimal form of a CAVE environment, four projectors are required to cover four surfaces of the room. In other examples such as [4] or [5], six projectors + eight Microsoft Kinect and five projectors + seven tracking cameras were respectively used to implement CAVE-like environments. Various restrictions sequentially stem from the large amount of hardware required for the CAVE-like environment. For instance, one just may have not enough budget to purchase all the devices. In addition, there are technical and physical challenges to install the entire system. Projectors and cameras have to be installed in a specific manner, so that they overlap and do not leave out any blind area, which all require both technical expertise and physical labor.

One solution to reduce the number of hardware required would be to utilize a steerable platform as did in Everywhere Displays [6] or Beamatron [7]. In both projects, a pan–tilt system was adopted to rotate the projector. The pan–tilt system allowed for augmenting rendered graphics on demand in 360° direction without using multiple projectors, which can remedy the budget limitation. If needed for the application, an RGB-D camera can be adopted to capture geometry information or track user’s interaction, comprising a projector-camera (pro-cam) unit. Then, the pro-cam unit is mounted on the steerable platform [7].

However, exploiting the 360° rotation capability does not always guarantee an immersive AR experience. To illustrate, in [7], the user had to stand in front of or in between the projection to interact with the system, since the only camera is rotated with the projector on the moving platform. If the user is out of the camera view, she has to manually call out command words to regain control of the system and orient it to herself. This not only limits the interaction area to the frontal area of the pro-cam system but also potentially causes blindness by the emitted projector light [8].

In this paper, we take a hybrid approach of both CAVE-like and the steerable systems to tackle the limitations present in either methods. To keep the number of the devices to a minimum, we utilize a pan–tilt pro-cam unit for immersive projection. To cover a large portion of the interaction area as CAVE does, we adopt an external camera in addition to the steerable pro-cam system, to track and interact with the user. Named PPAP, in short for Perspective Projection Augment Platform, the proposed system fully exploits the advantage of the actuated projector, by tracking the user’s viewpoint and automatically adjusting its orientation accordingly. Consequently, the system delivers seamless 360° projection AR with wide head-coupled perspective, which we call *actuated* perspective projection (Section 3.2). Our contributions to the literature can be summarized as follows:

1. We propose the design and configuration of a unique projection mapping system, where a stationary camera is used jointly with a pan–tilt motorized projector-camera unit.
2. We describe steps to calibrate and register multiple heterogeneous devices and control methods to consequently realize actuated projection mapping with such a system.
3. We demonstrate how the dynamically actuated projection with widened user-perspective benefit users in terms of correctly perceiving the spatial relationship of virtual objects.

## 2. Related Work

### 2.1. Immersive Projection System

Projection AR, unlike other HMD or mobile-based AR technologies, do not have a dedicated display, but uses surfaces of the real world as the medium and directly map graphic images onto them. This unique characteristic makes the projection AR to be easily scalable when compared to other ARs, as projection images can be stitched to form a unified enlarged screen. A traditional approach is to have a cubic space, and allocate a projection screen for each rectangular wall, typically front, left, right and floor. The distinct property of this approach is that it realizes a room-scale AR environment immersing users in the virtual world. The environment is often called a CAVE(-like) environment, named after the original CAVE [2].

Immersive projection systems like CAVE environments were widely adopted for training, entertainment, performance, education or office environments. To describe in detail, in [9], a CAVE-based simulation system was used to help drivers practice truck maneuvers and pallet operations. In RoomAlive [3], a more general, scalable method to calibrate and register multiple cameras and projectors using binary coded patterns was proposed. Authors of [10] utilized the immersive projection system to turn a room into virtual game space with a 360-degree all-around view. Reference [11] used projection AR to add design studio pedagogy to a computer science classroom. Augmented Studio [12] adopted projection mapping to teach physiotherapy students the mechanics of body movements by displaying anatomical information such as muscles and skeleton on the human body. In FUTUREGYM [13], four high performance projectors were installed on the ceiling to provide individual visual aids for children with special needs in a large-scale gym environment. Authors of [5] combined planar and 3D projection mapping to display informative contents of a dinosaur by augmenting the dinosaur itself and its surroundings. Immersive four-wall surrounding projection display was adopted also for an office environment where users can mirror their personal laptop’s display to discuss and collaborate with others [4].

**Figure 1:**
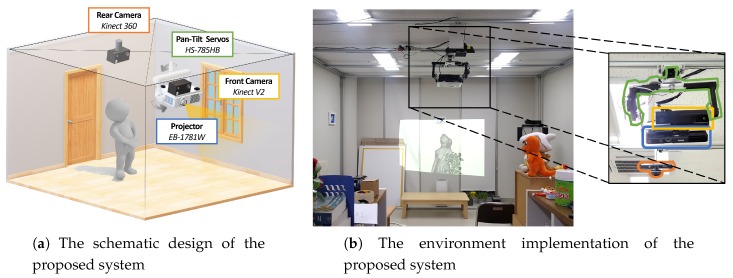
The environment setup of the proposed system. The front camera for capturing geometry data and the projector are mounted on the pan–tilt platform, all of which are installed in the middle of the room. The rear camera is installed in the far back to have a wide view of the environment to track and interact with the user. Each component in the images is color-coded—best viewed in color.

However, as versatile and effective as it may be, the immersive projection system has its own limitation. The primary reason is that, with multiple cameras and projectors, the system tends to be massive. As pointed out in [14], a large number of cameras are needed in proportion to the number of projectors to register all devices. Authors of [15] also noted that the CAVE environments imposes serious restrictions, such as size, mobility, economic expenses and so on. If the full-size environment is absolutely necessary, one has to accept these restrictions. However, in application scenarios where a single user or a master user is the main target, the system can focus on the target user’s view, and the rest of the environment is out of interest. For example, in Shadow Shooter [10], a single user wielded a bow and searched for an attacking virtual enemy. Thus, even though the system provided all-around virtual space, the projected image is displayed only at the front. Also in [5], a master user is assigned among users and the perspective projection of the virtual anatomy of a dinosaur model was provided to the master user. The follower users were then guided to the side of the master user to stand at good perspective viewpoints. In all these scenarios, an immersive all-surface projection system similar to [3] was used, but with immense expense and overuse of resources. Thus, we argue that, in such scenarios, such full-scale projection systems are unnecessary and can be substituted with a combination of a steerable projection-camera unit and a wide-area tracking camera, such as the proposed system (Figure 1). With the *actuated* projection mapping (Section 3.2), the proposed method was able to enhance users’ perception of spatial relationships of the virtual objects.The experiment design, result and its analysis are discussed in detail in Section 4.

### 2.2. Pan–Tilt/Steerable Controlled Platform

A pan–tilt platform or a steerable platform in general consists of two servo motors rotating horizontally and vertically. The two degrees-of-freedom grants the system it carries theoretically 360° FOV [16]. This dynamically enlarged FOV is especially beneficial to computer vision applications, as cameras can only obtain data along the optical axis directed at the time [17]. Thus, pan–tilt platforms were traditionally widely used for visual surveillance purposes [18]. For their ability to increase effective FOV of the device without actually adding extra hardware, they have been adopted to perform other tasks such as projection mapping, indoor scene reconstruction, or drone localization.

For projection mapping, Everywhere Displays [19] is well known for its concept of projection-augmenting multi-surface as interactive display in predefined positions with a projector and a rotating mirror. The concept was later reinforced in [20], where a camera was integrated with a rotatable projector to augment the environment with information, relatively to the marker’s position. A similar concept was also proposed in [21], where a Steerable Camera-Projector system was used to enable efficient man–machine interaction in multi-surface AR environments. It has evolved from previous systems, in that surfaces suitable for supporting projected interfaces were detected during an automatic environmental model building phase, without predesignations or explicit markers. In [7], a steerable projection system named Beamatron was introduced. The Beamatron consists of a RGB-D camera and a projector attached to a moving light platform that is capable of 360° pan–tilt rotations.

For indoor scene reconstruction, a pan–tilt-zoom camera was used in conjunction with stationary wide FOV depth cameras for capturing high-definition texture images in 3D reconstructing room-sized dynamic scenes [22]. For drone localization, controlled cameras—steerable cameras—were used used to register a flying drone in stereo-view, to increase the optical tracking volume without adding cameras.

It is notable that attempts to use a pan–tilt camera in conjunction with standard camera(s) for various applications have been proposed [22,23]. It is particularly notable that [23] and the proposed system share a common goal, which is to increase the volume of the space that a system can cover, whether it is an optical tracking system or a projection AR system. As we have described in Section 1, with one steerable pro-cam system such as [7], the user space is limited since the user not only has to stand in front of the system for interaction, and avoid the projection light at the same time to prevent self-occlusion and potential blindness hazard. In this paper, we propose a projection AR system, which integrates both a stationary camera and a pan–tilt projector-camera. The proposed system in Figure 1 yields substantial gain in both quality and quantity of the visually-tracked volume, enlarging interactive space and widening visible angle in an AR scene.

### 2.3. Perception/Presence in Graphics

Many research papers on computer graphics and human–computer interaction studied how humans perceive depth and spatial presence and what factors influence their performance. Various cues to deliver the sense of perception and presence in the virtual environment have been explored and studied in [24,25,26,27,28]. To summarize all the articles, there are four major factors that affect a human perception of spatial relationships in rendered graphics, namely perspective projection, motion parallax, shadow/shading and stereopsis. Based on the perspective geometry, perspective projection renders graphic scenes that are coupled with the user’s head, or viewpoint as we would normally see in the real world. Stereoscopic viewing exploits discrepancies in views of an object between two viewpoints, i.e., left and right eyes, to reflect the position and size of objects in 3D space. Motion parallax stands for difference in position or direction of object according to observation position or the viewer’s motion. Object shade and shadows provide spatial information about the relative position and size of objects in virtual space.

While some projection system provided a perspective stereoscopic view such as in a projected SAR tabletop setup [29], the proposed system at least in current setup do not render stereo graphics for two reasons. First, it eliminates any physical burden imposed on the user, even if that is a just a pair of 3D glasses. Since minimal user instrument is the strong advantage of the projection AR, we wanted to keep and maximize the merits. Second, previous research in projected SAR such as [27] reported that the users could perceive the spatial characteristics of virtual objects when they are projected without stereo. The other three major cues, perspective projection, shadow/shading and motion parallax, were implemented and can be provided with the proposed system.

To compensate for the absence of stereopsis in the system, we especially focus on the effect of motion parallax. The proposed system provides what we called *actuated* perspective projection. The system consistently tracks and anchors to the user’s point of attention and adjusts its orientation accordingly. As the result, the virtual view of the user is always rendered on the projected surface, delivering maximal viewing angle possible in an augmented scene (Figure 6). In this paper, we report that users were able to perceive remarkably better the spatial presence of the projected virtual object in the *actuated* projection (Section 4). Compared to the *stationary* projection, which is still user-perspective but not servo-actuated, the *actuated* perspective projection greatly increased the user’s viewing angle in the AR scene. We believe that the increased viewing angle made room for wider motion parallax, which enhanced users’ performance of perception and virtual spatial presence.

## 3. System Description

Figure 1 describes the configuration and environment setup for the proposed system. The system comprises a projector-camera unit motorized by two pan and tilt servo motors, and a rear camera, set up in a 3.7 × 4.0 × 2.25 m${}^{3}$ (width × depth × height) cubic space. An Epson EB-1771w projector (Long Beach, CA, USA) and Microsoft Kinect V2 (Redmond, WA, USA) were assembled as a projector–camera unit and mounted on a custom-made pan–tilting platform. The platform was operated by two Hitec HS-785HB servo motors (Poway, CA, USA), which were capable of multi rotations in a standard duty cycle. Thus, 360° projection-mapping was enabled with the pan–tilt platform. Microsoft Kinect 360 was adopted for tracking and measuring a user’s position and orientation. While the pan–tilt projector-camera unit and Kinect 360 were installed on the ceiling, the former was installed in the middle of the room while the latter was installed in the rear side of the room. This positioning decision was to maintain similar projection quality in any direction or area of the room, while enabling user-tracking in a wide region with a single camera.

Both front and rear cameras, Kinect V2 and Kinect 360, provide color and depth image streams. The front camera captures the data of the surface geometry that is to be projection-mapped and the rear camera tracks the user’s position and interaction in an AR scene. The user’s viewpoint and pose combined with the geometry data are employed to ray-cast the user’s line-of-sight and determine its center on the projection surface. The system computes optimal rotation angles of pan–tilt servos to fixate the projection center to the user’s view center. Then, the front camera and projector unit on the pan–tilt platform controlled with two HS-785HB servos is steered to provide 360° projection with the correct user’s perspective. As the system consistently adjusts its attitude with regard to the user’s position and perspective, the user’s view of the virtual object can be maximized and the user can be fully immersed in the AR scene. The overall process of the proposed system to control servo rotations is described in Section 3.2 and its effect in Section 4.

### 3.1. Projector-Camera-Servo Registration

In order to seamlessly integrate the user’s perspective in both the virtual view and the real world, cameras and the projector for vision capture and projection mapping should be solidly registered in a single common coordinate system. Thus, the problem of calibrating multiple device coordinate systems in a single common coordinate system arises. The calibration process is carried out in a pairwise manner, where all the calibrations between the front camera and the projector, between the front camera and the pan–tilt platform and between the pan–tilt platform and the rear camera should be taken into account. We solve for internal and external calibration parameters with multi-camera calibration [3] and pan–tilt rotation calibration [16] combined.

To bring all cameras and the projector to the common coordinate system, first there must be some portion of their views that are visible all among them. Therefore, the pan–tilt platform is rotated to a certain pose so that two front and rear cameras share a common view of some area of the projection. Generally, the pan servo is set to face the front and the tilt servo is set to match the pitch angle of the rear camera. In the proposed setup (Figure 1b), the pan rotation was set to 0° to face the wall and the tilt rotation was set to 45° with regard to the floor. We define the coordinate system of the front camera at this pose as the *reference* pose.

#### 3.1.1. Projector–Camera Calibration

**Figure 2:**
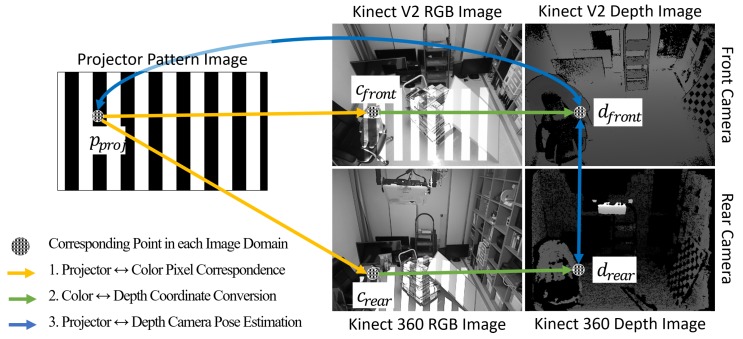
Computing pixel correspondences between cameras and a projector with gray code patterns. First, the pixel correspondences between the projector and both front and rear RGB-D cameras are computed. Then, corresponding points in the color images are transformed to depth image points. Finally, the depth image points are un-projected to 3D points, which are used to calibrate the projector and between front and rear cameras.

The *reference* pose is defined to establish a common coordinate system between the front and the rear camera. Using the gray code scanning [30], one can calibrate between two front and rear cameras, and between the front camera and the projector as well. Figure 2 describes the process for the multi-camera-projector calibration. First, gray code patterns are projected, which are captured by the front and rear cameras. The pixel correspondences are computed between the projector and each color camera. The color points of the corresponding pairs are converted to depth points, which are computed with color-depth calibration data from Kinect SDKs (Software Development Kit). Then, the depth points are un-projected into the 3D space. The 3D points combined with their corresponding points in the image domains are used to calibrate cameras and the projector using Zhang’s method [31]. We calibrate the projector as a reverse-camera model to acquire focal lengths $f_{x}$, $f_{y}$, principal points $c_{x}$, $c_{y}$ and its augmented 4×4 transform matrix $T_{proj\to front}$, with respect the *reference* pose of the front camera. The 4×4 translation and rotation matrices of the rear camera are also calibrated with respect to the *reference* pose. Using the calibrated parameters, a 3D point from the rear camera’s perspective is converted to a corresponding point in the *reference* coordinate as follows:

(1) $$\left[\begin{array}{c} P_{{ref}_{3\times 1}}\\ 1 \end{array}\right]=t_{rear\to ref}\;R_{rear\to ref}\left[\begin{array}{c} P_{{rear}_{3\times 1}}\\ 1 \end{array}\right].$$

#### 3.1.2. Camera-Servo Rotation Axis Calibration

**Figure 3:**
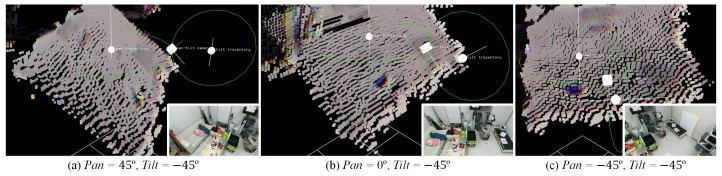
The rotation axis calibration results of the pan–tilt system. Pan and tilt trajectories were calculated (green circles). Note that the point cloud in the image is the projector that was captured from behind by the rear camera. Thus, the front camera was occluded, and its positions and poses were estimated in the figure labeled as the “pan–tilt camera” (white cubes). The images in the right-bottom corners are captured by the front camera to give the sense of its poses at the moment of the capture, during the rotation.

The front camera and the projector are fixed to the pan–tilt platform. Consequently, the transformations of their poses are dependent on the rotation of the pan–tilt platform. Thus, in order to accurately estimate the poses of the front camera and the projector, one has to model the movements and trajectories of the pan–tilt servos [16]. Following the steps described in the stated paper, a large checkerboard is placed in front against the wall. The rotation parameters of the pan–tilt platform are recovered in the *reference* coordinates, based on the corner points of the checkerboard. Denoting the pan and tilt rotation angles as α and β, the rotation parameters, which are positions and orientations of the rotation axes are respectively represented as $t_{pan}$, $R_{pan}\left(\alpha \right)$, $t_{tilt}$, $R_{tilt}\left(\beta \right)$. Based on the parameters, pan and tilt rotation trajectories can be modeled and the pose of the front camera on the rotation trajectories can be estimated (Figure 3). All combined, a point captured by the front camera is represented in the *reference* system as follows:

(2) $$\begin{array}{c} \left[\begin{array}{c} P_{{ref}_{3\times 1}}\\ 1 \end{array}\right]=F{\left(\alpha ,\beta \right)}_{front\to ref}\;\left[\begin{array}{c} P_{{front}_{3\times 1}}\\ 1 \end{array}\right],\\ \mathrm{where}\;F{\left(\alpha ,\beta \right)}_{front\to ref}=t_{pan}\;R_{pan}\left(\alpha \right)\;t_{pan}^{-1}\;t_{tilt}\;R_{tilt}\left(\beta \right)\;t_{tilt}^{-1}. \end{array}$$

*P* represents a point in 3D space. *R* and *t* are 4×4 matrices, respectively, for rotation and translation in 3D space. For simplicity, the term $t_{pan}\;R_{pan}\left(\alpha \right)\;t_{pan}^{-1}\;t_{tilt}\;R_{tilt}\left(\beta \right)\;t_{tilt}^{-1}$ is shortened as $F_{front\to ref}$.

### 3.2. Servo Control with User Perspective

**Figure 4:**
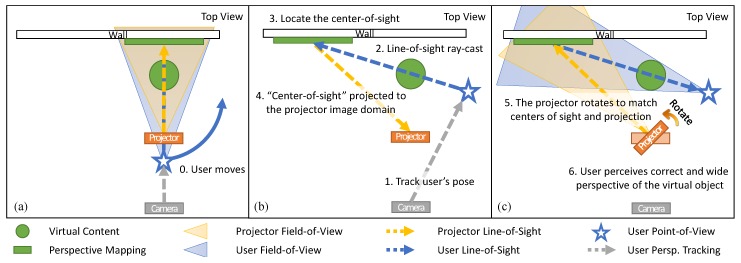
The illustration to demonstrate how servos are controlled to implement the *actuated* projection mapping. (**a**) the user’s position and perspective are tracked by the rear camera (gray arrow); (**b**) as the user inspects the virtual object (green circle), the user’s line-of-sight (blue arrow) is ray-casted to locate its view center on the surface geometry (green rectangle). The view center is projected (yellow arrow) on the projector’s image domain, and appropriate pan and tilt angles (α, β) are computed (Equation (3) and Figure 5); (**c**) the projector rotates to match its projection center and the user’s view center to augment more parts of the virtual object. The front camera is omitted for visibility.

**Figure 5:**
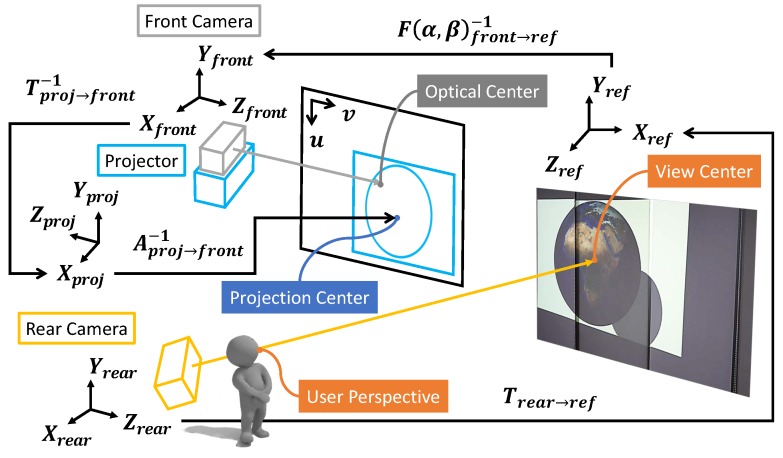
The illustration to describe and accompany Equation (3) and Figure 4. The user’s perspective is tracked by the rear camera and ray-casted to determine its view center on the surface in the *reference* coordinate. The view center coordinate is sequentially transformed to the *front* camera and the *projector* coordinate. Finally, the coordinate is projected onto the image domain of the projector to determine the corresponding point in the projection texture.

In a projection-based AR environment, the visible region of the AR scene to the user is eventually restricted, as there is a limit to the projector’s field-of-view. To overcome this limitation, the proposed system provides motorized projection mapping, which is coupled with the user’s perspective in the AR scene. The overall process is illustrated in Figure 4. First, the rear camera of the system consistently tracks the user’s position and orientation and computes the user’s line-of-sight. Next, the user’s line-of-sight is ray-casted to determine the user’s view center on the object-of-interest in the AR scene. The view center is then projected on the image domain of the projector. Given the 4×4 perspective matrix $A_{proj}$ and the pose matrix $T_{proj\to front}$ matrices of the projector, a point in 3D space is projected to a normalized 2D point on the projection image plane as follows (Figure 5):

(3) $$\begin{array}{c} s\left[\begin{array}{c} p_{{proj}_{2\times 1}}\\ 1_{{}_{2\times 1}} \end{array}\right]=A_{proj}\;T_{proj\to front}^{-1}\;F_{front\to ref}^{-1}\left(\alpha ,\;\beta \right)\;\left[\begin{array}{c} P_{{ref}_{3\times 1}}\\ 1 \end{array}\right],\\ \mathrm{where}\;A_{proj}=\;\left[\begin{array}{cccc} 2*f_{x}/w & 0 & -(2*c_{x}/w-1) & 0\\ 0 & -2*f_{y}/h & 2*c_{y}/h-1 & 0\\ 0 & 0 & -1 & 0\\ 0 & 0 & -1 & 0 \end{array}\right]. \end{array}$$

Here, *s* represents the scale factor in homogeneous coordinates. $f_{x},\;f_{y}$ are focal lengths and $c_{x},\;c_{y}$ are principal points of the calibrated projector. w,h are respectively width and height pixels of the rendered projection image. All combined, they consist of the perspective matrix $A_{proj}$ for the projector:

The pan–tilt platform is rotated to match the view center and the projection center, so that the user can perceive the augmented content in a more widened viewing angle. Then, the goal is to find the rotation angles α and β that minimize the following displacement error in Euclidean distance:

(4) $$\begin{array}{c} \arg\min_{\alpha ,\;\beta }∥\left[\begin{array}{c} p_{{proj}_{2\times 1}}\\ 1_{{}_{2\times 1}} \end{array}\right]{∥}_{2}^{2}. \end{array}$$

Points are calculated on the normalized image domain. Thus, the image center is at the origin, ${\left[0\;0\right]}^{⊺}$. Thus, we directly minimize the squared distance of the projected user’s view center from the origin. We solve this optimization problem based on the inverse kinematics approach, by extending the method of [16] for controlling servo motors to work with the projection point.

Since $A_{proj}$, $T_{proj\to front}$ and ${\left[P_{{ref}_{3\times 1}}\;1\right]}^{⊺}$ are all parameters with calibrated or given values, we can adopt the inverse kinematics algorithm in [16] by simply modifying the error and parameter vectors as $e=[p_{{proj}_{2\times 1}}]$ and $\theta ={\left[\alpha ,\;\beta \right]}^{⊺}$, respectively. By computing Jacobian matrix J(e,θ) of Equation (3) of the current pose, we can iteratively solve for two arguments α, β of $F_{front\to ref}^{-1}$.

### 3.3. Actuated Projection with Perspective Mapping

In the proposed system, the geometry information of the projection surface is acquired by the front camera and the user’s viewpoint and orientation are acquired by the rear camera. Microsoft Kinect V2 SDK’s skeleton tracking and PoseNet human pose estimation [32] were combined to respectively acquire the head position and eye positions. Then, the orientation vector is computed with triangulation of the three points. The user’s line-of-sight ray is casted from the head position, following the orientation vector’s direction. If eye positions are not available, the ear positions are used instead to determine the head orientation.

The user’s viewpoint and orientation are converted to *reference* points using Equations (2) and (1). The user’s perspective is then ray-casted to the surface geometry to compute the center of projection. The inverse kinematics algorithm [16] with the center point of Equation (3) determines the pan–tilt rotation angles α and β. With user’s perspective projection matrix $A_{user}$ formed as [33], Algorithm 1 is established to realize actuated perspective projection.

**Algorithm 1** Actuated Perspective Projection    **for** each *frame*
**do**  Receive 3D points $P_{front}$ and the user’s viewpoint $E_{rear}$  Compute pan–tilt angles α and β with Equation (3)  Configure $P_{ref}$ and $E_{ref}$ with Equations (2) and (1)  Configure the perspective matrix $A_{user}$ from $E_{rear}$  Render the view from $A_{user}$ [33] as texture *projTex*  Set rendered result as *projTex* for projective texture  **for** each $P_{ref}$ in geometry **do**        Compute texture coordinate *projCoord* $=A_{user}\;P_{ref}$        Map *projTex* onto geometry with *projCoord*


## 4. User Experiments

**Figure 6:**
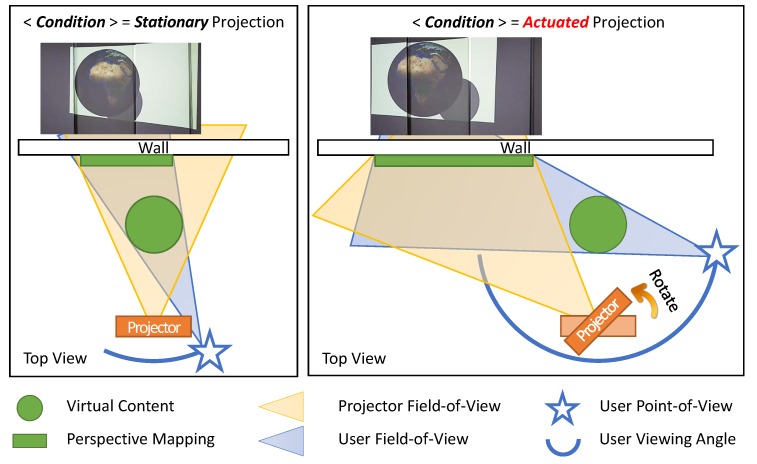
The schematic comparing the *stationary* and *actuated* projection. Note that both projection mappings are user-perspective rendered. *Actuated* projection mapping with pan–tilt servos greatly widens the viewing angle in a virtual scene, as more regions of the Middle East Asia region are revealed before the earth goes out of the projection range. Front and rear cameras are omitted for visibility.

As illustrated in Figure 6, the *actuated* perspective projection enabled by the proposed system provides the user with a much wider view in the projected augmented environment, when compared to the *stationary* projection environment such as [27]. Note that the *stationary* projection does not mean that the projection itself is static. It is the projector that is stationary, and the rendering is still coupled to the user’s head. To evaluate the effectiveness and usefulness of the proposed *PPAP* system, we conducted a user experiment that can demonstrate its characteristic, which is the widened viewing angle in a projection AR scene.

In monoscopic projection AR, the augmented virtual scene is mapped onto the real world surface, without the stereopsis effect that boosts the spatial perception. In such cases, various spatial cues can aid subjects with perceiving spatial relationships in an AR scene, including perspective projection, shading, shadow mapping and motion parallax [24,26]. In the experiment, we focused on examining the effectiveness of increased motion parallax on a user’s sense of the virtual object’s spatial presence in the monoscopic perspective projection AR environment. Specifically, we examined whether users can perceive spatial relationships of the projected object as spatial in mid-air, rather than as merely projected on the surface, and which factors affected their perception.

We recruited 11 participants aged from 25 to 31 (mean = 27.1 years, standard deviation = 1.8 years). All participants completed the experiment and were asked to give their subjective opinions on their experience with the proposed system and the experiment. The total session including the experiment and the interview took approximately 30 min to complete.

### 4.1. Experiment Design

**Figure 7:**
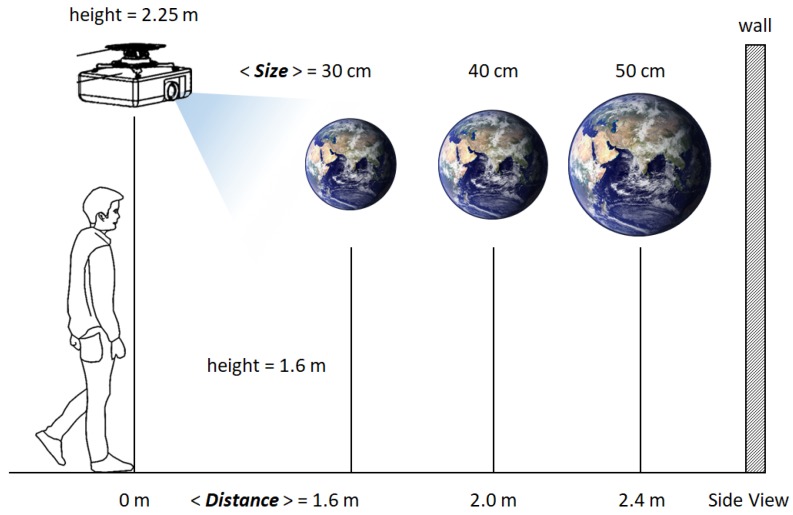
The illustration of the experiment configuration. Users rate the **Size** and **Distance** (in the image) of the projected virtual object, the globe. The size and distance of the object are projected twice in two conditions (Figure 6).

To measure how users perceive the spatial presence of a virtual object, the participants were asked to rate the size and distance of the virtual objects, similar to the experiment design of [27]. As illustrated in Figure 7, a globe was chosen as the test object and was projected with three different sizes (30 cm, 40 cm and 50 cm radii) and three different distances (1.6 m, 2.0 m and 2.4 m away) virtually hovering at 1.6 m height. The back wall on which virtual objects were projected was approximately 2.8 m away from the origin. The sizes and distances were designed so that the objects with different size*distance configurations may be perceived as roughly the same by the users. For example, the largest yet farthest object and the smallest yet closest object appear as if they are of roughly the same scale. These confusions were intentionally designed to minimize obvious answers and robustly evaluate the effect of the experiment conditions on user’s spatial perception. We imposed much harsh conditions (less discernable size*distance configurations) than those of [27] to better manifest factors that influence correct spatial perception.

The main purpose of the experiment was to analyze the effect of the *actuated* perspective projection, where the projector unit rotates itself to match the user’s view center and the projection center so that users can view larger parts of the virtual object before it goes out of the projection range. Thus, in addition to different size*distance combinations, the experiment was conducted with two different conditions, namely *stationary* and *actuated* (Figure 6). In the *stationary* condition, the pan–tilt head remained stationary throughout the experiment. In the *actuated* condition, which is the proposed system, the pan–tilt head continuously steered to match the centers of the user’s view and the projection.

In [27], two different conditions “with physical markers” and “without physical markers” were used to evaluate the effectiveness of the projected SAR system proposed in the stated paper. In this paper, no physical markers were used throughout the experiment. The rationale behind this decision was two-fold. First, we assumed that users could perceive spatial relationship in an AR scene without the aid of physical markers, as no statistical importance was found between “with” and “without” conditions in [27]. Second, we wanted to observe the sole influence of the projection condition, which is whether the projection is *stationary* or *actuated*, on the user’s spatial perception performance. Without any other affecting factors, the effect of the proposed system could be well manifested.

Through this experiment, we aimed to verify the following hypotheses:

**H1.**  The participants are able to perceive spatial representation of the virtual objects, which are projection-augmented.

**H2.**  The participants are able to rate the size and distance more correctly if they can view wider range of the augmented object.

**H3.**  The actuation of projection can enhance the depth perception, which is attenuated as the projection surface is distanced.

To summarize, the experiment was designed to have 18 configurations (Size (3) × Distance (3) × Condition (2)). For each participant, three trials of the 18 configurations were performed, which yielded total 54 ratings. We note that the experiment was designed as within-subjects. Thus, participants were randomly partitioned into two half groups and the presentation order of *stationary* and *actuated* conditions were counterbalanced between groups.

### 4.2. Experiment Procedure

Before the experiment began, the participants were briefed about the experiment including the system description, experiment goals, their rights, limitations and so on. The descriptions were given both verbally and in literal forms on their information sheets.

The participants were then asked to stand at the origin point, which we defined as the perpendicular foot of the projector-camera unit to the floor. They were given a session to familiarize with the system and practice before the actual experiment began. In the practice session, nine size*distance configurations of the earth globe projection with correct answers were presented to the participant, in the stationary projection condition. The rationale behind this decision was that the proposed actuated projection was a superset of the stationary projection and the test group. Thus, it would be only fair for users to familiarize with the system in the stationary projection condition. No time limit was imposed on the practice sessions, yet all practice sessions took less than two minutes to complete.

As noted earlier, the presentation order of *stationary* and *actuated* conditions was counterbalanced between two half groups, in order to minimize the learning effects caused due to the ordering. For both groups, participants carried out two conditions in a sequence. Between conditions, participants were given a short break while the coordinator gathered the results. For each condition, the presentation orders of the projection conditions were randomized beforehand, and fixed throughout the experiment for all participants.

In the experiment, the participants rated the size and distance of the the projected virtual earth globe verbally, using terms “small”, “medium” and “large” for the size and “near”, “middle” and “far” for the distance. For each size*distance configuration, a 15-second time limit was imposed on the participants. The time limit was relatively loose when compared to that of [27] (5 s). This design decision was made to reflect the characteristic of the proposed system, where the effective FOV of the projection could be enlarged by orienting the projector in accordance with the participants’ viewpoint (Figure 6). Thus, the participants were given ample amount of time to, and were encouraged to, actively move around and investigate various regions of the virtual earth globe.

The participants were directed to give their ratings when they felt confident. They were waited until the allotted time expired. Then, the projection was turned off (rendered black) and participants were urged to report their ratings as quickly as possible. The reported ratings were recorded by the coordinator. Then, the trial with the next configuration was carried out. The time for participants to make their decisions was not measured, as we rationalized that the 15-second time limit was generous enough for participants to make thorough decisions and thus their response time held low significance.

### 4.3. Results and Analysis

#### 4.3.1. Hypotheses H1 and H2

**Figure 8:**
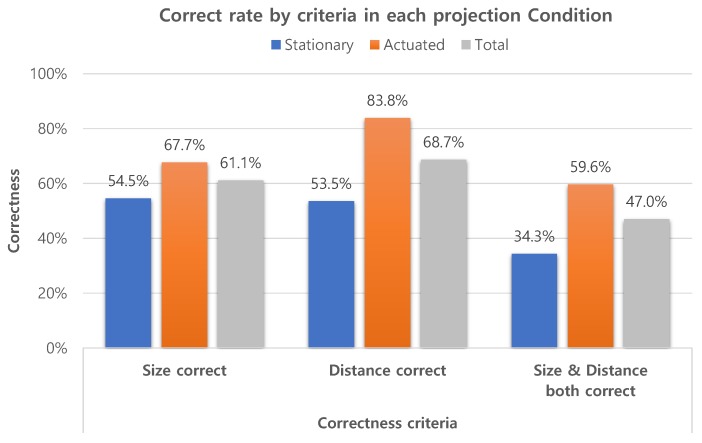
The ratios of correct ratings, determined by three different criteria: size, distance, and both size and distance.

Figure 8 summarizes the experiment results on how users accurately perceived the sizes and distances of the virtual object. For the hypothesis H1, we only investigate the total results in the graph. The participants were able to correctly rate 61.1% of the size variations and 68.7%of the distance variations. If we assess the overall correctness, that is, when both the size and distance are correct, the participants were able to correctly rate 47.0% of all size*distance combinations. Since there were nine size*distance combinations of the virtual object, a random guess would have 11.1% (1 out of 9) chance of being correct. The overall correct rate of 47.0% is significantly higher than the random guess probability 11.1%. These results confirm our first hypothesis H1 that participants can correctly perceive the presented spatial relationship of the virtual objects, which are projection-augmented by the proposed system.

We constructed confusion matrices, predictions vs. ground truth, from the participants’ ratings of size and distance. We found that only 4.7% of the total ratings missed by more than one option, such as mistaking a “near” distance as “far”. The low percentage of missed-by-more-than-one-option answers is important in two things. First, it is another piece of evidence for hypothesis H1 in which participants are able to perceive virtual objects’ presence correctly, at least indirectly. Second, it lays the groundwork for encoding participants’ responses into a binary scale, either “correct” or “incorrect”. The binarization of responses allows us to analyze the performance results using binomial regression, which simplifies the interpretation and analysis of H2 and H3. Since the experiment was designed as a within-subject study and categorical responses were collected, the repeated measures logistic regression should be employed [27]. We used Generalized Estimating Equations (GEE) of IBM SPSS Statistics v25 (Armonk, NY, USA) to analyze for correctness of user’s ratings. The correlation between the experiment configuration parameters—size, distance and projection condition—and the binary correctness variable were computed. Wald Chi-Square ($\chi^{2}$) was calculated to evaluate the statistical significance of each predictor to the model.

Three variables, Size, Distance and Condition, were chosen as predictors to analyze the overall correctness and tested for correlations on the model. We set significance level as α = 0.05, Thus, we found something statistically significant and rejected the null hypothesis, if the associated *p*-value was p ≤ 0.05. The condition, whether the projection was *stationary* or *actuated*, was found to have the statistically high significance ($\chi^{2}$ = 12.431, df = 1, p < 0.001), while the size ($\chi^{2}$ = 2.139, df = 2, p = 0.343) and distance ($\chi^{2}$ = 5.201, df = 2, p = 0.074) were found otherwise.

Figure 8 summarizes correct rates in each projection condition. The overall correctness results grouped by condition were 34.3% with the *stationary* projection, and 59.6% with the *actuated* projection. The statistical significance of the projection condition, and the difference in the overall correctness between projection conditions firmly support hypothesis H2, that the participants are able to rate the size and distance more correctly when the viewing angle of the augmented object is wider. These results also support hypothesis H1. Since different results were produced when the projection condition was changed, it would be rational to conclude that the participants’ answers were neither random, nor memorized from their training sessions, but the results of spatial perception.

During the subjective feedback session, participants reported that they were more comfortable and immersed in the *actuated* perspective projection condition, and thus it was more easy to notice the spatial relationship of virtual object, which coincides with the quantitative assessment. We conjecture that the actuated perspective projection widened the viewing angle, and consequently results in greater motion parallaxes and more shadow mapping effects. Combined with the statistical analysis results, we conclude that the proposed system (*actuated* projection) improved the spatial perception and presence of the projection mapped AR contents.

#### 4.3.2. Hypothesis H3

Previously, several research papers on projection AR have reported that the projection quality degrades as the projection surface is distant, and users find augmented objects less present as a result [27,34]. Hypothesis H3 was designed to test whether the proposed *actuated* perspective projection can mitigate the effect of the projection quality degradation, and enhance virtual objects’ spatial presence. Thus, we analyzed subsets of result data and investigated in detail how the projection condition affected users’ depth perception by the distance.

**Figure 9:**
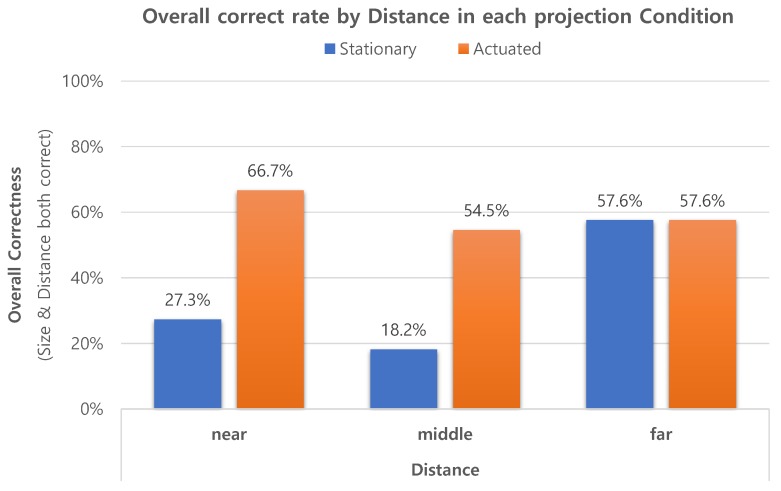
The ratios of correct ratings, categorized by distance in each condition, *stationary* and *actuated*, and their total results.

Statistical analysis showed that the Distance factor was significant with both the *stationary* projection ($\chi^{2}$ = 9.695, df = 2, p = 0.008) and the *actuated* projection ($\chi^{2}$ = 5.992, df = 2, p = 0.049). However, distributions of two subset results were found to be quite different from each other, as shown in Figure 9. In the *stationary* condition, the correct rates were 27.3%, 18.2% and 57.6%, respectively, for “near”, “middle” and “far”, the experiment configuration and result distribution of which are similar to [27]. On the contrary, in the *actuated* condition, the correct rates were 66.7%, 54.5% and 57.6%, respectively, for “near”, “middle” and “far” in the *actuated* condition, showing the opposite distribution, where the better performance was achieved as the distance was closer.

The discrepancies in correct rates between *stationary* and *actuated* conditions indicate clear improvements of the participants’ spatial perception in “near” and “middle” distances, while they performed the same in the far distance. We conjecture that the *actuated* projection mapping of the proposed system negated the degradation of the virtual presence in projection AR, and boosted the participants’ spatial perception, even if the virtual object was far detached from the projection surface.

**Figure 10:**
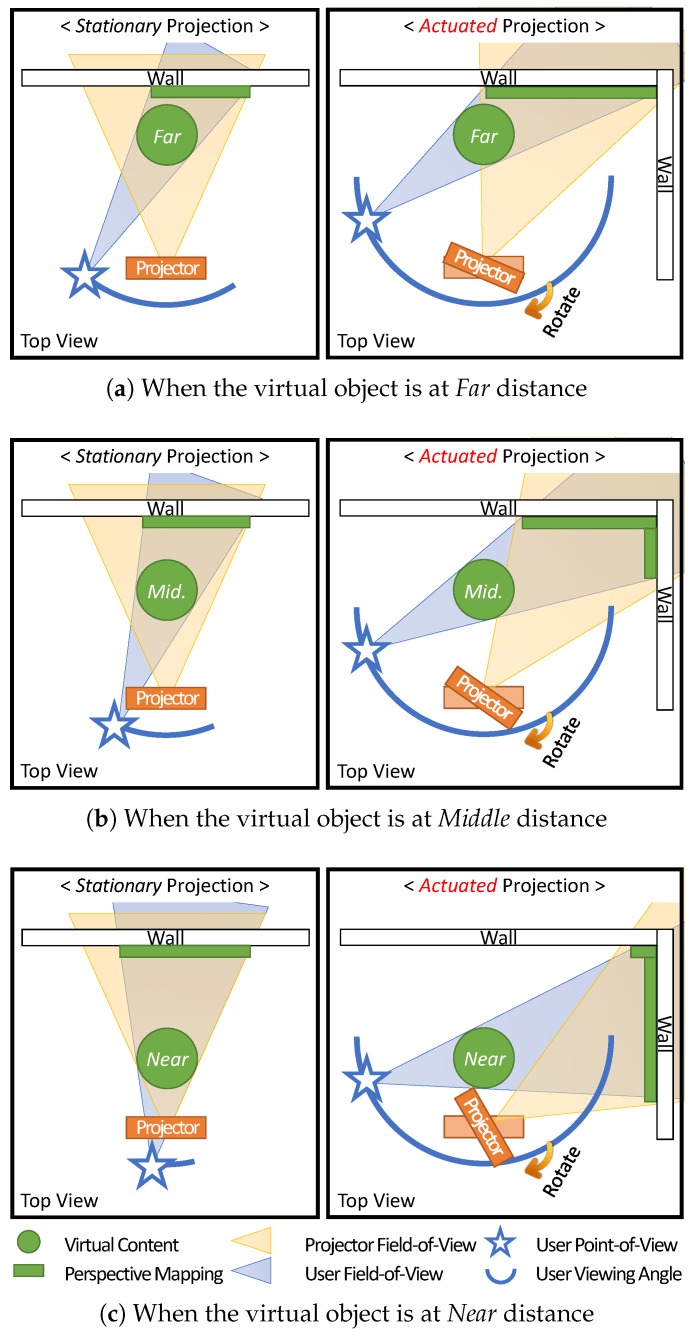
Changes in the viewing angle of the virtual object of varying distances, depending on the *stationary* or *actuated* projection conditions. In *stationary* perspective projection, the user’s viewing angle gradually diminishes as the virtual object is placed close to the user. However, in *actuated* perspective projection, the projector can rotate to widen the viewing angle, to the extent of the pan-tilt servos’ rotation bounds. For visibility, the front and rear cameras are omitted.

The accompanying rationale is illustrated in Figure 10. In the *stationary* projection, the visible angle of the virtual object becomes greatly limited as it is located closer to the user. This is because the projector’s FOV is fixed, and thus the visible area of the virtual object is limited depending on the projection distance. On the contrary, in the *actuated* projection, the projector can be rotated, which increases the effective FOV of the projection. Thus, regardless of the virtual object’s position, provided that the user’s perspective view of the object is within the bound of pan–tilt servo rotations, the projector can augment the object with correct spatial presence and perspective.

### 4.4. Discussion and Limitations

Monoscopic projection is well known for its wider viewing angle and for its freeing users from the instrumentation, which are two major limitations in other types of AR, especially in the case of mobile AR and HMD. However, it is also reported that the projection of the virtual content that was far from the surface degraded in quality [34], and it resulted in users’ under-performance in perceiving spatial presence of virtual objects as they were distanced from the surface [27].

We believe that the proposed system can boost the strength and mitigate the weakness of the monoscopic projection AR, since all three hypotheses of the *actuated* projection are validated through user experiments. The *actuated* projection increases the viewing angle in an AR scene over the physically limited field-of-view of the projector, to the scope of 360° by steering and coinciding the display region of interest with that of the user. Moreover, the *actuated* projection not only delivers the spatial presence of virtual objects in ideal conditions, i.e., the projection is close to the surface, but also preserves it comparably in harsh conditions where the projection is distanced from the surface. Since improved spatial perception leads to enhanced spatial presence, we believe that the proposed system can ultimately promote immersion in an AR environment.

While all is promising, there is still room for improvement in the proposed method. As the proposed *actuated* projection anchors to a specific user’s viewpoint, it may not be ideal for hosting multiple users in a shared virtual environment. In such a scenario, we believe a couple of strategies from the literature can be applied to support multiple users and provide good perspective in the shared AR environment.

Firstly, the “master” user strategy of [5] could be adopted in the proposed system. The paper introduced the concept of the “master” user for projection mapping in a CAVE-like environment. In the environment, dinosaur-related contents such as anatomy and habitat were presented to users, via projection mapping. As virtual contents were projection-mapped onto a real physical model of a dinosaur, self-shadowing was inevitable. To reduce visible shadows, the position of the “master”, chosen among viewers, was correlated with the projectors’ frustums. Other users were were guided to an *ideal* viewpoint with good perspective positions, using color-coded circles with an additional arrow. Since the *actuated* projection is optimized for a single user’s perspective, the “master” user strategy can be directly applied to the proposed method for multi-user scenarios.

Secondly, the Dynamic Zoning approach proposed in [35] could be adopted in the proposed system. The Dynamic Zoning approach tackles the problem of rendering multiple users’ views in a surrounding virtual environment. Particularly, in a specific case when two users are looking in the same direction, the scene is rendered from the “democratized” point between users’ head positions. If applied to the proposed system, the wide-area camera can track multiple users’ viewpoints, and average the viewpoints. Rendered from midpoint, the equal experiences can be provided to all users.

### 4.5. Possible Application

The proposed system is capable of tracking a user’s viewpoint, and dynamically augmenting virtual objects with actuated projection. Thus, the proposed system has the advantages in projection AR environments, where a solo or key user is allocated with a focused view of the AR scene. In such environments, the proposed system may be adopted to replace the existing hardware, or to extend the function of the current installation. We believe the characteristic of the proposed system raises many potential possibilities and applications for the projection mapping.

For example, Gallery Invasion [36], which was a projection-based art installation in an art gallery, was implemented with a projector with a moving mirror to provide three-sided wall projection mapping. Although the projection was immersive and magical, the viewpoint in the rendered graphics remained fixed throughout the play, limiting the chance of participatory experiences. This is because there was no equipment for user tracking, and the system had to assume that the user always stands at a predesignated point.

The used hardware is similar to the proposed system in that they all support 360° projection mapping. However, the proposed system is also capable of capturing 360° geometry information and tracking users’ positions and perspectives in a wide area. Thus, if the proposed system had been adopted instead, we believe the viewer could have experienced the projected contents in full, as they were able to move and engage freely in the AR environment.

## 5. Conclusions

In this paper, we introduced PPAP, a projection augment platform for perspective projection with dynamic actuation of the pan–tilt platform. We proposed the design and configuration for the PPAP system, where a stationary camera is used jointly with a pan–tilt motorized projector-camera unit. We implemented actuated user-perspective projection mapping, in which the pan–tilt motorized projection system continuously steers itself to match the user’s view center of the projection AR scene to that of the projection. Through user studies, we verified that the actuated perspective projection helps users better understand the spatial relationships in an AR scene in terms of depth perception. As future work, we would like to develop real world applications where the actuated perspective projection would be found useful, and evaluate its benefit on users.
